# Prognostic MicroRNA Fingerprints Predict Recurrence of Early-Stage Hepatocellular Carcinoma Following Hepatectomy

**DOI:** 10.7150/jca.79593

**Published:** 2023-02-05

**Authors:** Victor Chun-Lam Wong, Ming-In Wong, Victor Ho-Fun Lee, Kwan Man, Kevin Tak-Pan Ng, Tan To Cheung

**Affiliations:** 1 OncoSeek Limited, Hong Kong Science and Technology Parks, Hong Kong Special Administrative Region, People's Republic of China; 2 Department of Clinical Oncology, Queen Mary Hospital, LKS Faculty of Medicine, The Hong Kong Special Administrative Region, People's Republic of China; 3 Department of Surgery, Queen Mary Hospital, LKS Faculty of Medicine, The Hong Kong Special Administrative Region, People's Republic of China

**Keywords:** Hepatocellular carcinoma, miRNA fingerprints, Liquid biopsy, Machine learning, HCC diagnosis, HCC prognosis, hepatectomy

## Abstract

**Purpose:** This study aims to develop liquid biopsy assays for early HCC diagnosis and prognosis.

**Methods:** Twenty-three microRNAs were first consolidated as a panel (HCCseek-23 panel) based on their reported functions in HCC development. Serum samples were collected from 103 early-stage HCC patients before and after hepatectomy. Quantitative PCR and machine learning random forest models were applied to develop diagnostic and prognostic models.

**Results:** For HCC diagnosis, HCCseek-23 panel demonstrated 81% sensitivity and 83% specificity for identifying HCC in the early-stage; it showed 93% sensitivity for identifying alpha-fetoprotein (AFP)-negative HCC. For HCC prognosis, the differential expressions of 8 microRNAs (HCCseek-8 panel: miR-145, miR-148a, miR-150, miR-221, miR-223, miR-23a, miR-374a, and miR-424) were significantly associated with disease-free survival (DFS) (Log-rank test p-value = 0.001). Further model improvement using these HCCseek-8 panel in combination with serum biomarkers (i.e. AFP, ALT, and AST) demonstrated a significant association with DFS (Log-rank p-value = 0.011 and Cox proportional hazards analyses p-value = 0.002).

**Conclusion:** To the best of our knowledge, this is the first report to integrate circulating miRNAs, AST, ALT, AFP, and machine learning for predicting DFS in early HCC patients undergoing hepatectomy. In this setting, HCCSeek-23 panel is a promising circulating microRNA assay for diagnosis, while HCCSeek-8 panel is promising for prognosis to identify early HCC recurrence.

## Introduction

Hepatocellular carcinoma (HCC) is a highly fatal cancer, accounting for nearly 830,000 deaths every year worldwide 1. Traditionally, alpha-fetoprotein (AFP) is the most common serological biomarker for HCC detection. However, AFP detection is known to have a low specificity issue and low positive predictive value (PPV) 2-4. Therefore, more reliable biomarkers for both HCC diagnosis and prognosis are needed.

MicroRNAs are endogenously expressed small non-coding RNA first discovered by Ambros and Ruvkun groups in 1993. Circulating microRNAs have been under the spotlight for diagnostic and prognostic applications because of their functions in various liver diseases such as viral hepatitis, alcoholic or non-alcoholic liver diseases, liver fibrosis, and cirrhosis 5,6. Since miRNA has been reported to outperform AFP for HCC diagnosis 6-8, circulating microRNA is one of the emerging liquid biopsy technologies to complement AFP for HCC screening and prognosis 7-9.

The advent of machine learning enables us to analyze multi-dimensional data for clinical applications 10,11. In HCC, Chaudhary et al applied machine learning to analyze the genetics of the tissue biopsies for clinical application 12. However, whether machine learning can translate the molecular genetic profile from the liquid biopsy for HCC prognosis still needs to be examined. Previously our group compared different machine learning strategies such as regression-based (logistic regression), non-regression-based (neural network), and ensemble machine learning models (random forest and gradient boosting) using liquid biopsy miRNA profile for HCC diagnosis. We identified random forest as a promising model for identifying HCC. In this study, we aim to apply the random forest model for HCC prognosis. To do so, we analyzed multi-dimensional data, including microRNA expressions, dynamic change in miRNA levels before and after hepatectomy, and traditional serum biomarkers.

This study sought to build a liquid biopsy microRNA (miRNA) assay for HCC diagnosis and prognosis. We began by consolidating 23 miRNAs (HCCseek-23 panel) essential for HCC development. Subsequently, we tested the diagnostic power of HCCseek-23 panel for early HCC diagnosis. After that, we narrowed down the miRNA panel to eight signature miRNAs (HCCseek-8 panel) based on their association with patient survival. Ultimately, we developed an integrated prognostic model with the HCCseek-8 panel in combination with the traditional serum biomarkers AST, ALT, and AFP.

## Methodology

### Patient enrolment criteria

This is a retrospective study with all the data prospectively collected in a patient database. Patients with clinically diagnosed resectable HCC were included. All the patients had CT/MRI typical features of HCC. Patients who were diagnosed with HCC were discussed in the multidisciplinary meeting with oncologists, radiologists, and surgeons. Patients who were considered technically resectable by minimally invasive surgery and with good liver function reserved were included. Patients with the extrahepatic disease, Child C liver cirrhosis, and severe pre-existing medical conditions were excluded. A total of 103 HCC patients treated with hepatectomy at Queen Mary Hospital between January 2006 and October 2012 were included in this study (Table 1). All blood samples were obtained after written informed consent. Eighty-one patients were diagnosed with stage I and 17 patients were diagnosed with stage II based on The Hong Kong liver cancer (*HKLC*) staging system.

### Random forest model development

Random forest is a modified version of the bagging tree-based machine learning technique. It avoids overfitting by sampling the samples and features randomly at first. Initially, the samples (i.e. HCC patients) are randomly subset to multiple training sets by bootstrapping. Then, in each training set, a decision tree is established by randomly extracting a part of the features from the features (i.e. microRNAs, AST, ALT, AFP) until an optimal solution is reached. The final prediction result is the classification result from the majority vote among all the decision trees in the random forest model. For the first section of developing an HCC diagnostic model, 55 intermediate-stage HCC patients (Barcelona Clinic Liver Cancer system stage B) and 33 healthy individuals reported in our previous paper were used 13. The microRNA expression qPCR results were split into a training dataset (n= 70) and a test dataset (n= 18), followed by random forest model development. After that, the HCCseek-23 random forest model's diagnostic performance was evaluated using 196 serum samples from the early HCC patients treated with hepatectomy (Table 1). For the second section of developing an HCC prognostic model, as mentioned in the enrolment criteria section, a total of 103 HCC patients treated with hepatectomy at Queen Mary Hospital between January 2006 and October 2012 were included in this study. The samples were collected before (B1) and after the surgical operation (B2). Samples from five patients were removed due to poor quality (i.e. more than 10 miRNAs showed Ct value higher than 35. The clinical information of the subjects is summarized in Table 1. During the model development, we first classified the patients into two groups based on the median survival data (i.e. 86.2 months for overall survival (OS) and 58.81 months for disease-free survival (DFS)). The long and short OS group has a mean OS of 135.9 and 47.3 months, respectively, while the long and short DFS group has a mean DFS of 110.3 and 19.7 months, respectively. After labeling the samples with the survival classification, we split the dataset (n=98) into the training dataset (n=68) and test dataset (n=30). Subsequently, the survival group classifications and the microRNA expression levels were subjected to random forest model development, followed by validation with Log-rank and Univariate Cox proportional hazards analyses.

### Sample preparation and miRNA isolation

To ensure the quality of the serum sample, a two-step centrifugation was applied. First, we removed the blood cells with low-speed centrifugation, followed by high-speed centrifugation to remove residual impurities. A volume of 300 uL serum sample was transferred to a new 1.5mL centrifuge tube and the protein debris was precipitated with high-salt buffer for 1 min at room temperature. The supernatant containing RNA was then precipitated by isopropanol, followed by RNA purification using a column-based Silica membrane technology. After washing the columns with ethanol-based buffer, RNase-free H_2_O was added into the column, followed by 1-minute room temperature incubation and RNA elution. The eluted RNA was subjected to PolyA tailing reaction with PolyA polymerase and ATP and incubated for 60 minutes at 37°C, followed by 5-minute deactivation at 70°C. Subsequently, cDNA synthesis was performed by adding oligo-dt adapter primer, miRNA-specific forward primer, reverse transcriptase, and incubate for 20 minutes at 42°C, followed by 85°C incubation for 5 minutes 13.

### MicroRNA analysis

Roche Light-Cycler 480 was applied for qPCR reaction. The Ct values for microRNA were generated with the 2nd derivative maximum of fluorescence curve. For the microRNA with no Ct value after calculation, a Ct value of 45 was filled to facilitate further model development. The Ct values were converted to fold differences by the equation 2^(-ΔCt) normalized to the endogenous expression level of miR-451a.

### Statistical analysis and data visualization

Clinicopathologic and genomic variables were tested for their effect on the survival probabilities of patients; the effect of these prognostic factors were estimated by the Kaplan-Meier method and compared between survival subgroups by Log-rank test and univariate Cox proportional hazards analyses. The effect size of univariate survival analysis was estimated based on p-values and hazard ratio with 95% confidence intervals (95% CI); p-values < 0.05 were interpreted as statistically significant associated with survival outcome. A hazard ratio >1 indicates that the variable associated with increased risk of death, while hazard ratio< 1 indicates the variable associated with decreased risk of death; hazard ratio= 1 means that variable have no effect on the length of survival. Lifelines 0.25.7 in python (version 3.8.3) was used for statistical analyses. Student's T test were performed using SciPy package in python environment, the effect size was estimated by the difference between two means divided by pooled standard deviation. Univariate Cox proportional hazards model and the Log-rank test were analyzed using lifelines.statistics package in python. The results were visualized in Kaplan-Meier curves and Cox proportional hazards model hazard ratio plot using matplotlib.pyplot and lifeline package in python.

## Results

### Establishing HCCseek-23 microRNA panel

**Figure 1:**
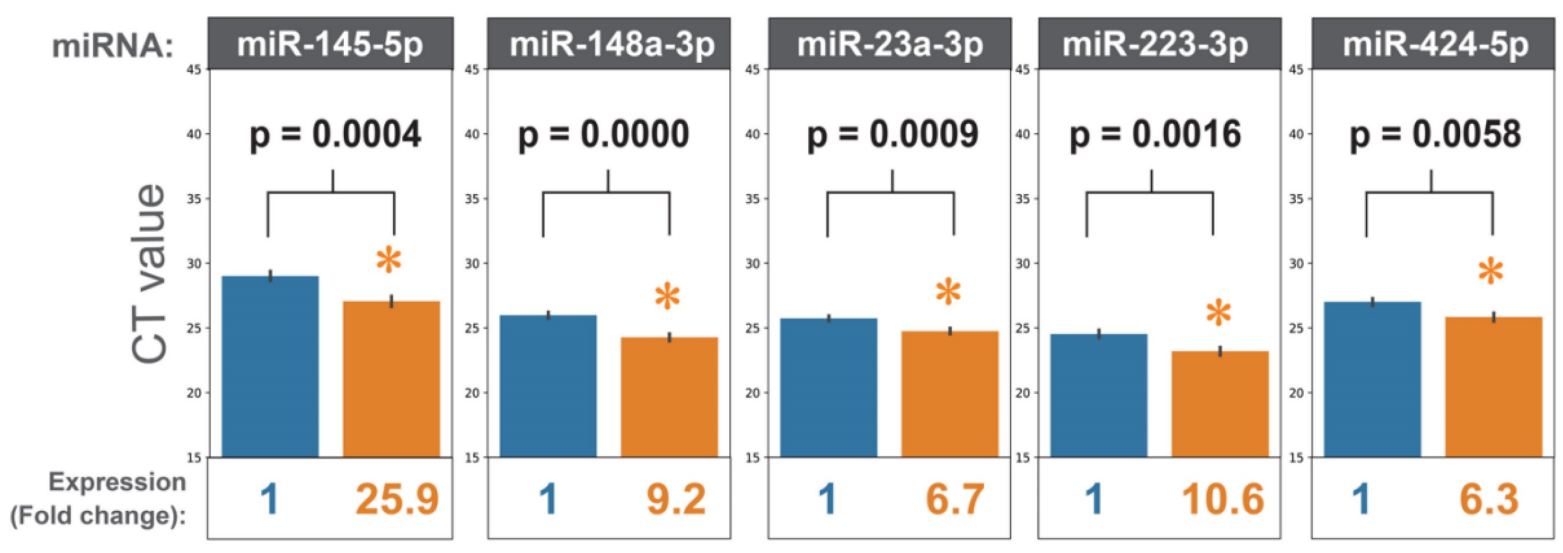
MiRNAs expressions before and after hepatectomy. When comparing the miRNA expressions before surgery (blue bars) and after surgery (orange bars), eighteen miRNAs showed statistically significant results. For simplicity, five miRNAs are shown (i.e. miR-145-5p, miR-148a-3p, miR-223-3p, miR-23a-3p, miR-424-5p). Expressions of all the miRNAs can be found in supplementary Figure 1. * indicates p-values<0.05 in Student's t-test.

To design an HCC-specific microRNA panel, we reviewed the literature focusing on microRNAs' functions in HCC development. Twenty-three microRNAs were selected based on their reported functions in regulating cancer hallmarks in HCC (Supplementary Table 1). Quantitative PCR (qPCR) was performed to detect the expression levels of these 23 microRNAs (HCCseek-23 panel) in the serum samples taken from 103 stage I and II HCC patients before and after hepatectomy. After quality control filtering, 98 HCC patients were available for analysis. Seventeen microRNAs (miR-122-5p, miR-125a-5p, miR-125b-5p, miR-145-5p, miR-148a-3p, miR-191-5p, miR-192-5p, miR-214-3p, miR-22-5p, miR-223-3p, miR-23a-3p, miR-30c-5p, miR-320d, miR-365a-3p, miR-423-5p, miR-424-5p, miR-574-3p) were significantly up-regulated after surgery (p-value < 0.05, Figure 1, Supplementary Figure 1, Supplementary Table 2-3). As expected, the negative control microRNA miR-451 showed no difference comparing pre- and post-surgical operations (Supplementary Figure 1).

### HCCseek-23 miRNA random forest model can identify early HCC

Before we develop an HCC prognostic model, we attempted to test if the HCCseek-23 microRNA panel could identify early-stage HCC patients. To do so, we applied the random forest model we reported previously 13 to this HCCseek-23 miRNA panel (detailed in the methodology section). As expected, the HCCseek-23 random forest model provided good performance for identifying stage I and II HCC patients (n=196, 81% sensitivity, 83% specificity, and 0.79 AUC) and identifying early-stage AFP-negative HCC patients (n= 45, 93% sensitivity and 83% specificity, Supplementary Table 4). Collectively, these results suggested miRNA's clinical utility for early HCC diagnosis.

### HCCseek-23 miRNA random forest model is not associated with patient survival

Next, we test if the HCCseek-23 random forest model is associated with patient survival. To do so, we applied the Log-rank test and univariate Cox proportional hazards analyses. In the Log-rank test, no significant association was found between the predicted HCC probability and the OS and DFS of the early-stage HCC patients (n=98) (p-values are 0.09 and 0.25; Supplementary Table 5). In univariate Cox proportional hazards analysis, we separated the patients into two categories (long-term and short-term survival groups) based on the median OS and DFS for evaluation. Again, no significant association was found between the predicted HCC probability and the survivor classification (Supplementary Figure 2-3). To sum up, although the HCCseek-23 random forest model can identify early-stage HCC patients, it is unsuitable for HCC prognosis. It is necessary to narrow down the microRNA panel specifically for prognosis purposes.

### Identification of signature miRNAs for OS and DFS prognosis

To tailor-make a microRNA panel for early-stage HCC prognosis, we first narrowed down our microRNA list by the Log-rank and Cox proportional hazards analysis. To do so, we calculated the differential microRNA expression pattern comparing post-operation versus pre-operation time points (B2-B1). These differential expressions, together with the expression data at both pre-operation (B1) and post-operation (B2) time points were subjected to the Log-rank and Univariate Cox proportional hazards tests. Eight microRNAs (HCCseek-8 panel i.e. miR-145, miR-148a, miR-150, miR-221, miR-223, miR-23a, miR-374a, miR-424) showed significant association with DFS in either Log-rank test or univariate Cox proportional hazards analysis (Table 2). When analyzing OS, four microRNAs (HCCseek-4 panel i.e. miR-125a, miR-223-3p, miR125b, miR-150) showed significant association (Table 2). The KM-curves, p-values, and cumulative hazard ratio plots were shown in Supplementary Figures 4**-**17**.** In summary, we narrowed down the microRNA panel to 8 microRNAs and 4 microRNAs for further prognostic model development.

### Signature miRNA panel predicts DFS but not OS

**Figure 2:**
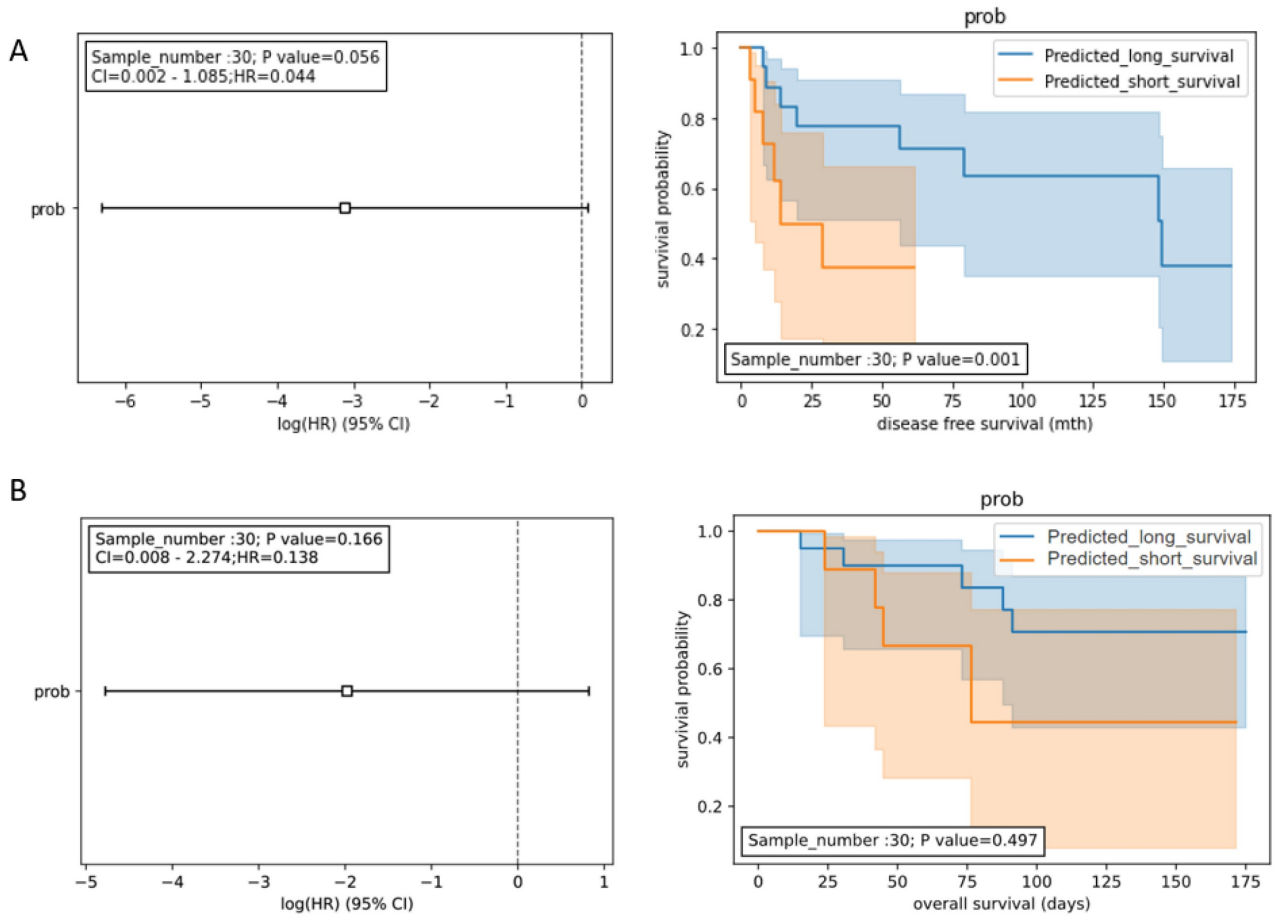
Survival analyses for the signature miRNA prognostic model. (A) Prognostic models developed from 8 miRNA panel (HCCseek-8). The log-rank test showed significant association between the predicted survival probability score and the DFS (p-value = 0.001), while the univariate COX proportional hazards model showed marginal association (p-value=0.056). (B) Prognostic models developed from 4 microRNA panel (HCCseek-4). The log-rank test and the univariate COX proportional hazards model showed no significant association between the predicted survival probability score and the OS. The hazard ratios are shown in the forest plots (left panel). The orange lines and blue lines in the Kaplan-Meier curve (right panel) shows the survival probabilities of the long-term survival group and the short-term survival group predicted by the prognostic models.

To develop a prognostic model for DFS, the expression data of the 8 miRNA panel (HCCseek-8) shown in Table 2 were input into the Random Forest model. During the model development, the early-stage HCC patients (n=98) were split into the training dataset (n= 68) and the testing dataset (n= 30) using the 70:30 ratio. To evaluate the prognostic performance of the HCCseek-8 miRNA model, we analyzed the predicted survival probability with Log-rank and Univariate Cox proportional hazards tests. A significant association was observed between the predicted survival probability and the patient survival (p-value = 0.001) in Log-rank test, meanwhile, marginal significance was observed in Univariate Cox proportional hazards analysis (p-value = 0.056, Figure 2A). To develop a prognostic model for OS, the expression data of the 4 miRNA panel shown in Table 2 were input into the Random Forest model. However, no association between the predicted survival score and patient survival was observed (Log-rank p-value = 0.497; Cox analysis p-value = 0.138) (Figure 2B). To sum up, the signature miRNAs HCCseek-8 panel is correlated with DFS, suggesting a potential application for predicting DFS. Further model improvement is needed for HCC prognosis.

### Integrating HCCseek-8 and serum biomarkers for developing HCC prognosis model

**Figure 3:**
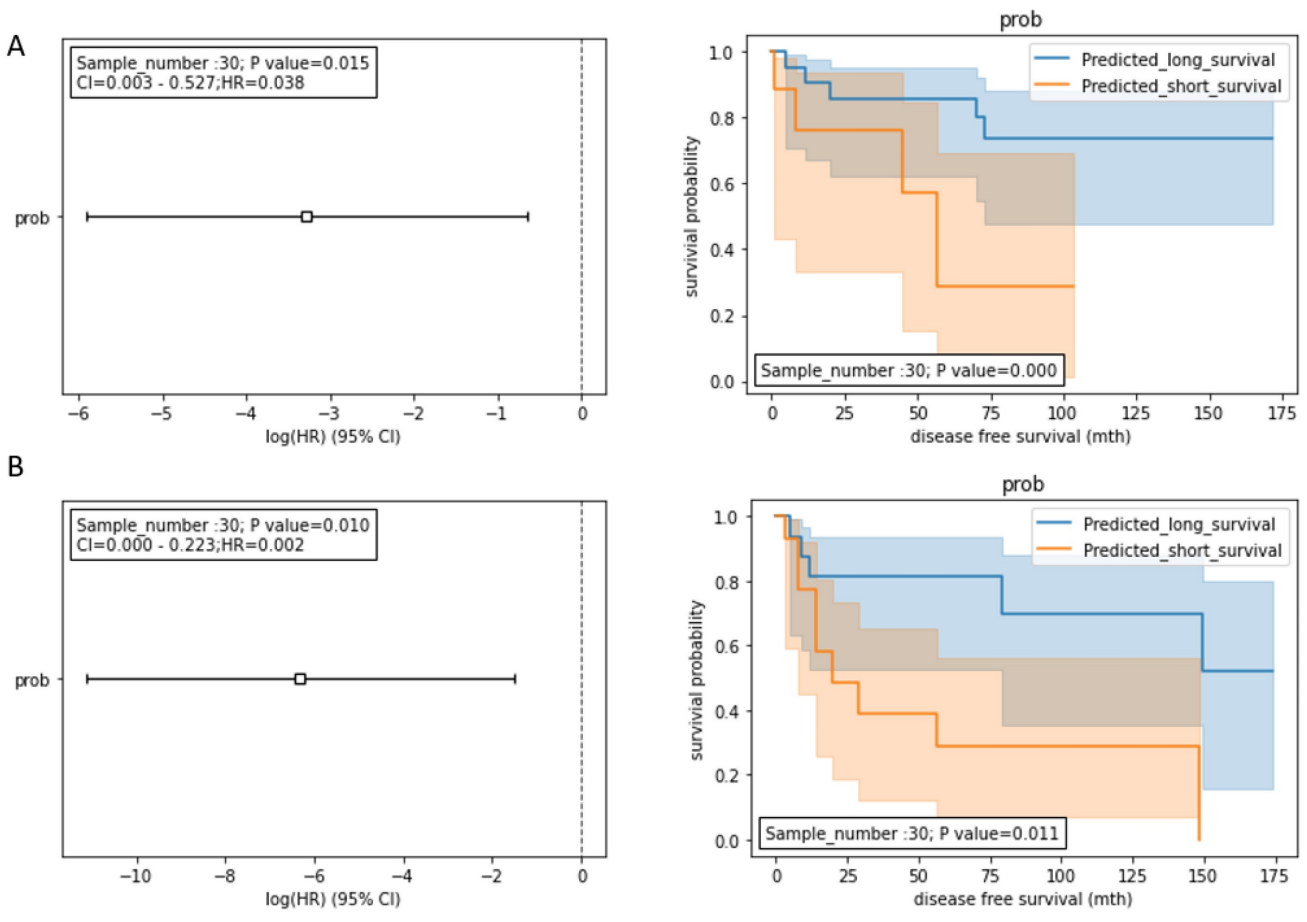
Survival analyses for the integrated models. (A) Prognostic models integrating HCCseek8- panel, AFP, and AST. The log-rank test and the univariate COX proportional hazards model showed significant association between the predicted survival probability score and the DFS (log-rank p-value = 0.0003, Cox p-value = 0.015, HR (95%CI) = 0.038 (0.003-0.527)). (B) Prognostic models integrating HCCseek-8 panel, AFP, AST, and ALT. The log-rank test and the univariate COX proportional hazards model showed significant association between the predicted survival probability score and the DFS (log-rank p-value = 0.011, Cox p-value = 0.010, HR (95%CI) = 0.002 (0.000-0.233)). The orange lines and blue lines in the Kaplan-Meier curve indicate predicted long-term survival group and predicted short-term survival group, respectively.

To further improve the prognostic model for predicting DFS, we integrated the model with the traditional serum biomarkers (AST, ALT, and AFP). AST and ALT are liver function-damage indicators, while serum AFP is a serological biomarker for HCC diagnosis 14. These routinely used biomarkers could be meaningful for introducing an additional data dimension to improve our prognostic model. Therefore, we inputted the miRNA, AST, ALT, and AFP data into the random forest model development. During the model development, the stage I and II HCC patients treated with hepatectomy (n=98) were split into the training dataset (n= 68) and the testing dataset (n= 30). We tested two integration models: 1) HCCseek-8 + AFP + AST; and 2) HCCseek-8 + AFP + AST +ALT. The “HCCseek-8 + AFP + AST +ALT” model showed a more significant association with DFS (Log-rank p-value= 0.011, Cox p-value= 0.010, HR= 0.002, 95% CI: 0.000-0.233), compared to “HCCseek-8 + AFP + AST” model (Log-rank p-value= 0.0003, Cox p-value= 0.015, HR= 0.038, 95% CI: 0.003-0.527) (Table 3, Figure 3A , Figure 3B). Notably, when we remove HCCseek-8 from the model development, the negative control model “AFP+AST” and “AFP+AST+ALT” did not show any significant association with DFS (Table 3). This illustrated the essential role of the microRNA panel in predicting DFS in patients undergoing hepatectomy. Taken together, we provided solid evidence demonstrating the prognostic power of integrating novel HCCseek-8 microRNA panel and traditional serum biomarkers for prognosis in early HCC patients treated with hepatectomy.

## Discussion

In summary, this study highlighted four key findings: 1) We developed an HCC diagnostic model using the expression profile of twenty-three miRNAs (HCCseek-23 panel), which demonstrated 81% sensitivity and 83% specificity for identifying early-stage HCC patients; 2) HCCseek-23 panel also exhibited 93% sensitivity for identifying AFP-negative HCC; 3) We further identified eight microRNAs (HCCseek-8 panel) that was significantly associated with DFS in the Log-rank test (p-value = 0.001), suggesting a potential application for HCC prognosis; 4) When integrating HCCseek-8 panel and serum biomarkers, a significant association between the prediction result and DFS was found in both Log-rank test (p-value = 0.011) and Cox proportional hazards analysis, illustrating an outstanding performance for predicting DFS in early-stage HCC patients undergoing surgical operations.

Functional roles of the HCCseek-8 microRNAs in the progression of HCC have been previously documented 15-24. The miR-145-5p 15, miR-148a-3p 16, miR-150-5p 17, miR-223-3p 19, and miR-424-5p 22 were found to be down-regulated in HCC. The down-regulated expressions appear to potentiate cancer cell migration and invasion in HCC patients. These microRNAs serve as HCC suppressors by targeting transcription factors and oncogenes associated with cancer cell growth, migration, and metastasis. For instance, miR-145-5p targets the ARF6 pathway to inhibit invasion and metastasis. Downregulation of these miRNAs is known to promote HCC invasion and metastasis 15-17,19,22. Besides regulating invasion and metastasis, the loss of miR-145-5p, miR-150-5p, and miR-223-3p expressions has been identified to promote HCC proliferation 15,17,19. The miR-148a-3p is critical in controlling hepatic differentiation by regulating c-Met oncogene 16. In addition, the decreasing level of miR-148a-3p in the blood of HCC patients had an inverse relationship with the profibrogenic cytokine TGF-β, associated with the progression from cirrhosis to HCC, and linked to poorer survival outcomes 24. MiR-221-3p 18, miR-23a-3p 20, and miR-374a-5p 21 are involved in tumor cell proliferation, genomic stability, and growth suppressor evasion in HCC.

Although some microRNAs in the HCCseek-8 panel have been linked to HCC detection and survival 5,7,18,23,24, analyzing multi-dimensional data could improve the prediction model 10-12. In this study, we integrated multi-dimensional data such as the microRNA expressions, dynamic change of miRNA levels before and after surgery, and the traditional serum biomarkers for the prognosis of early HCC patients undergoing hepatectomy (Supplementary Figure 18). In addition, we demonstrated the diagnostic performance of the microRNA panel for identifying early-stage HCC patients. Although a large-scale and multi-center investigation is still needed to translate these models into clinical application, this study demonstrated promising results for further model development in the future.

## Supplementary Material

Supplementary figures and tables.

## Figures and Tables

**Table 1:** Clinical characteristics of HCC patients treated with hepatectomy

98 patients (196 serum samples in total)				
Sex	Male		Female	
	75		23	
AFP group	AFP positive		AFP negative	
	53		45	
HCC stage	Stage I		Stage II	
	81		17	
	Mean	Std	Min	Max
Age(year)	60.94	10.5	28	82
Body weight(kg)	63.71	12.5	31.5	107
Body Height(cm)	162.65	7.63	144	179
Pre-operation AST(u/L)	40.58	18.63	16	109
Pre-operation ALT (u/L)	42.47	24.49	12	142
Pre-operation platelet (10^9/L)	162.83	51.31	66	288
Pre-operation AFP (ng/ml)	930.46	5458.68	1	53430
Overall survival (month)	91.91	51.01	4.14	180.04
Disease-free survival (month)	65.03	54.7	0.72	177.51

AFP>= 20ng/mL= AFP positive group; AFP< 20ng/mL= AFP negative group

**Table 2:** Eight miRNA panel selected for DFS prognosis and Four miRNA panel selected for OS prognosis

Eight miRNA panel			
**MiRNA**	**Univariate Cox analysis** **(p-value)**	**Hazard Ratio** **(HR)**	**Log-rank** **(p-value)**
**miR-145-5p (B2-B1)**	0.71	0.97	0.02*
**miR-148a-3p (B1)**	0.54	0.00	0.04*
**miR-150-5p (B2)**	0.64	0.01	0.01*
**miR-150-5p (B2-B1)**	0.47	1.00	0.01*
**miR-221-3p (B1)**	0.84	0.00	0.04*
**miR-223-3p (B1)**	0.41	0.00	0.00*
**miR-23a-3p (B2-B1)**	0.05*	1.02	0.46
**miR-374a-5p (B1)**	0.04*	0.00	0.53
**miR-374a-5p (B2-B1)**	0.02*	1.05	0.32
**miR-424-5p (B2-B1)**	0.96	1.00	0.01*
**Four miRNA panel**			
**miR-125a-5p (B1)**	0.94	0.03	0.05*
**miR-125b-5p (B1)**	0.61	1.00	0.04*
**miR-150-5p (B1)**	0.33	1.00	0.00*
**miR-223-3p (B1)**	0.25	0.00	0.01*

Note: * p-value ≤ 0.05; B2-B1: differential microRNA expression pattern comparing post-operation versus pre-operation time points; B1: pre- operation; B2: post-operation

**Table 3:** Log-rank and Cox analyses for the integrated prognostic models and negative control

Model	Biomarkers	Cox (p-value)	Log rank (p-value)	HR (95% CI)
**Integrated Model**	HCCseek-8 + AFP + AST	0.015*	0.0003*	0.038 (0.003-0.527)
	HCCseek-8 + AFP + AST + ALT	0.010*	0.011*	0.002 (0.000-0.233)
**Negative control Model**	AFP + AST	0.657	0.664	0.405 (0.07-2.128)
	AFP + AST + ALT	0.716	0.475	0.292 (0.036-2.354)

Note: * p-value<0.05
