# Supplementary material for: Prognostic MicroRNA Fingerprints Predict Recurrence of Early-Stage Hepatocellular Carcinoma Following Hepatectomy
Source: J Cancer. 2023 Feb 5;14(3):480–9. doi: 10.7150/jca.79593 (PMC9969587; doi:10.7150/jca.79593)
Supplement: Supplementary file 1 — Supplementary figures and tables. [file jcav14p0480s1.pdf]

Supplementary Table 1: 23 microRNAs and their reported function in regulating cancer hallmarks in HCC

miRNA										
miRNA	Sustaining Proliferative Signaling	Evading Growth Suppressors	Avoiding Immune Destruction	Enabling Replicative Immortality	Tumor Promoting Inflammation	Activating Invasion / Metastasis	Inducing Angiogenesis	Genome Instability	Resisting Cell Death	Deregulating Cellular Energetics
150-5p	**[25]**					**[17]**				
574-3p		[26]								
125b-5p	**[27]**	[28]				[27]			[28]	
191-5p						[29]				
23a-3p		[20]	[30]							
145-5p	**[31]**	[32]				[31]			[33]	
125a-5p						[34]			[35]	
214-3p	**[36]**	[37]				[36]				
192-5p				[38]					[39]	
320d	**[40]**									
26a-5p	**[41]**					[41]			[41]	
365a-3p										
148a-3p				[42]	[43]	[44]	[44]			
423-3p		[45]					[46]			
486-5p	**[47]**					[47]				
22-5p										[48]
223-3p	**[49]**								[49]	
423-5p		[45]	[50]						[51]	
374a-5p	**[52]**					[52]		[52]		
221-3p						[53]		[18]		
30c-5p					[54]					
424-5p	**[55]**	[56]	[57]			[55]	[58]			
122-5p	**[59]**					[59]				
451a	**[60]**					[60]				

Supplementary Table 2: Summary of the miRNA expressions detected in qPCR for the samples collected before surgery (B1)

Before surgery(n=98)	mean	std	min	max
miR-122-5p	24.16	2.96	19.17	45.00
miR-125a-5p	28.93	2.39	24.08	38.00
miR-125b-5p	28.59	2.81	24.40	45.00
miR-145-5p	29.01	3.76	24.54	45.00
miR-148a-3p	25.99	2.36	21.86	35.37
miR-150-5p	28.65	2.88	22.03	45.00
miR-191-5p	27.76	2.92	23.89	45.00
miR-192-5p	27.64	1.83	23.06	33.98
miR-214-3p	27.59	2.68	24.53	45.00
miR-22-5p	29.31	1.90	25.15	36.65
miR-221-3p	27.48	2.41	24.28	38.00
miR-223-3p	24.53	2.78	20.48	38.00
miR-23a-3p	25.75	1.96	23.11	38.00
miR-26a-5p	27.60	2.30	24.85	38.00
miR-30c-5p	25.50	1.69	22.66	32.96
miR-320d	31.45	1.79	28.58	38.00
miR-365a-3p	28.01	1.55	23.53	34.17
miR-374a-5p	29.15	2.54	25.09	45.00
miR-423-3p	30.01	3.11	24.78	45.00
miR-423-5p	27.67	2.53	23.22	38.00
miR-424-5p	27.02	2.72	22.80	38.00
miR-486-5p	23.75	2.81	19.62	38.00
miR-574-3p	29.92	1.97	26.42	38.00
miR-451a	18.26	2.94	13.91	38.00
preop AST (u/L)	40.58	18.68	16.00	109.00
preop ALT (u/L)	42.47	24.55	12.00	142.00
preop platelet (10^9/L)	162.83	51.44	66.00	288.00
preop AFP (ng/ml)	930.46	5472.73	1.00	53430.00
overall survival (mths)	91.91	51.14	4.14	180.04
disease free survival (mth)	65.03	54.85	0.72	177.51

Supplementary Table 3: Summary of the miRNA expressions detected in qPCR for the samples collected before surgery (B2)

After Surgery(n=98)	mean	std	min	max
miR-122-5p	21.91	2.47	15.15	33.35
miR-125a-5p	27.90	2.29	24.40	38.00
miR-125b-5p	27.56	3.82	20.17	45.00
miR-145-5p	27.06	3.87	21.52	45.00
miR-148a-3p	24.28	2.77	17.98	38.00
miR-150-5p	28.08	3.14	22.03	45.00
miR-191-5p	26.94	2.61	23.77	38.00
miR-192-5p	25.86	2.03	19.57	33.56
miR-214-3p	26.75	1.83	23.76	35.63
miR-22-5p	28.34	2.12	23.17	36.17
miR-221-3p	26.87	2.64	24.02	38.00
miR-223-3p	23.20	3.03	19.10	38.00
miR-23a-3p	24.75	2.16	21.99	35.95
miR-26a-5p	27.23	2.35	24.75	38.00
miR-30c-5p	24.68	1.84	20.27	33.78
miR-320d	30.57	2.04	24.40	38.00
miR-365a-3p	26.71	1.78	21.68	35.46
miR-374a-5p	28.71	2.32	25.61	38.00
miR-423-3p	29.23	2.94	25.59	45.00
miR-423-5p	26.87	2.67	22.96	38.00
miR-424-5p	25.85	3.12	22.87	38.00
miR-486-5p	23.63	3.11	18.91	38.00
miR-574-3p	28.42	1.91	23.59	38.00
miR-451a	18.37	3.05	14.32	35.46
preop AST (u/L)	40.58	18.68	16.00	109.00
preop ALT (u/L)	42.47	24.55	12.00	142.00
preop platelet (10^9/L)	162.83	51.44	66.00	288.00
preop AFP (ng/ml)	930.46	5472.73	1.00	53430.00
overall survival (mths)	91.91	51.14	4.14	180.04
disease free survival (mth)	65.03	54.85	0.72	177.51

Supplementary Table 4: HCCseek-23 Random Forest model performances, sensitivity and specificity for HCC diagnosis

Model	Sample size in training and testing dataset	Accuracy(std) in testing dataset	Sample size in validation dataset	Accuracy(std) in validation dataset	AUC in ROC analysis	Sensitivity	Specificity	Sensitivity for AFP(-) HCC (n=45)
HCCseek-23 Random Forecast Model	88 (HCC, n=55; Healthy, n=33)	0.75 (0.040)	196 (Stage I and II patients treated with hepatectomy)	0.887 (0.010)	0.79	0.81	0.83	0.93

Supplementary Table 5: Performance of HCCseek-23 panel random forest model for HCC prognosis

	Univariate Cox proportional hazards test p-values	Hazard Ratio(HR)	Log rank p-values
**Prognostic for DFS**	0.61	2.03	0.25
**Prognostic for OS**	0.61	2.02	0.09

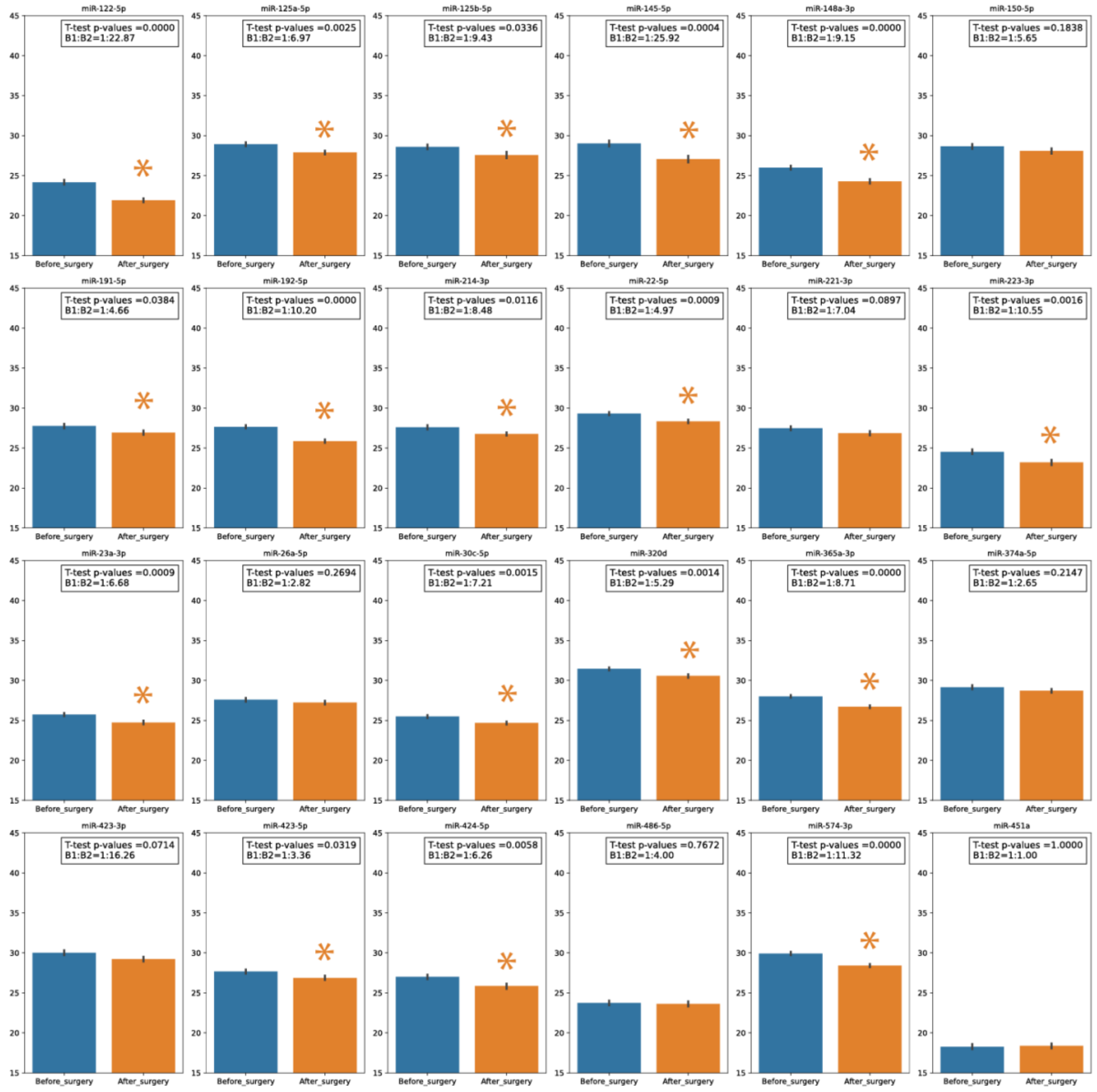

Supplementary Figure 1. The expressions of 23 miRNAs before and after hepatectomy. The change of expressions in eighteen miRNAs are statistically significant when comparing the expression levels before surgery (blue bars) and after surgery (orange bars) (i.e. miR-122-5p, miR-125a-5p, miR-125b-5p, miR-145-5p, miR-148a-3p, miR-191-5p, miR-192-5p, miR-214-3p, miR-22-5p, miR-223-3p, miR-23a-3p, miR-30c-5p, miR-320d, miR-365a-3p, miR-423-5p, miR-424-5p, miR-574-3p). The bars shows the Ct values obtained in qPCR. The fold-change of expressions are shown as B1:B2 at the right-hand comer of each subplot. B2 indicates miRNA expressions after surgery while B1 indicates miRNA before surgery. * indicates p-values<0.05 in Student's t-test.

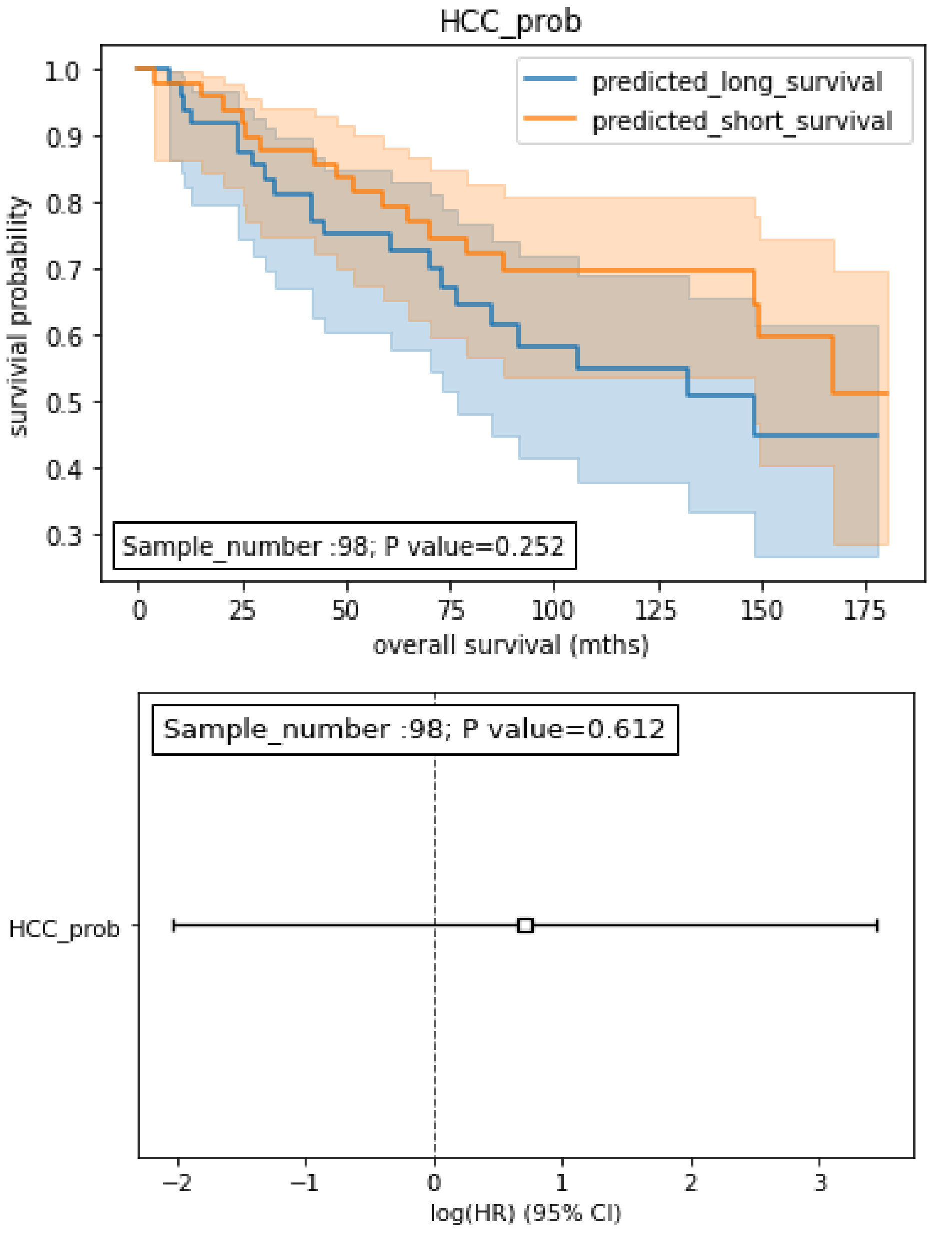

Supplementary Figure 2: Performance of HCCseek-23 panel for HCC overall survival (OS) prognosis as analyzed by log-rank and univariate Cox hazards tests. Kaplan-Meier survival curves and Univariate Cox shows no significant differences between the predicted HCC probability (cut-off > 0.5) and OS (log-rank p-value= 0.252, cox p-value – 0.612).

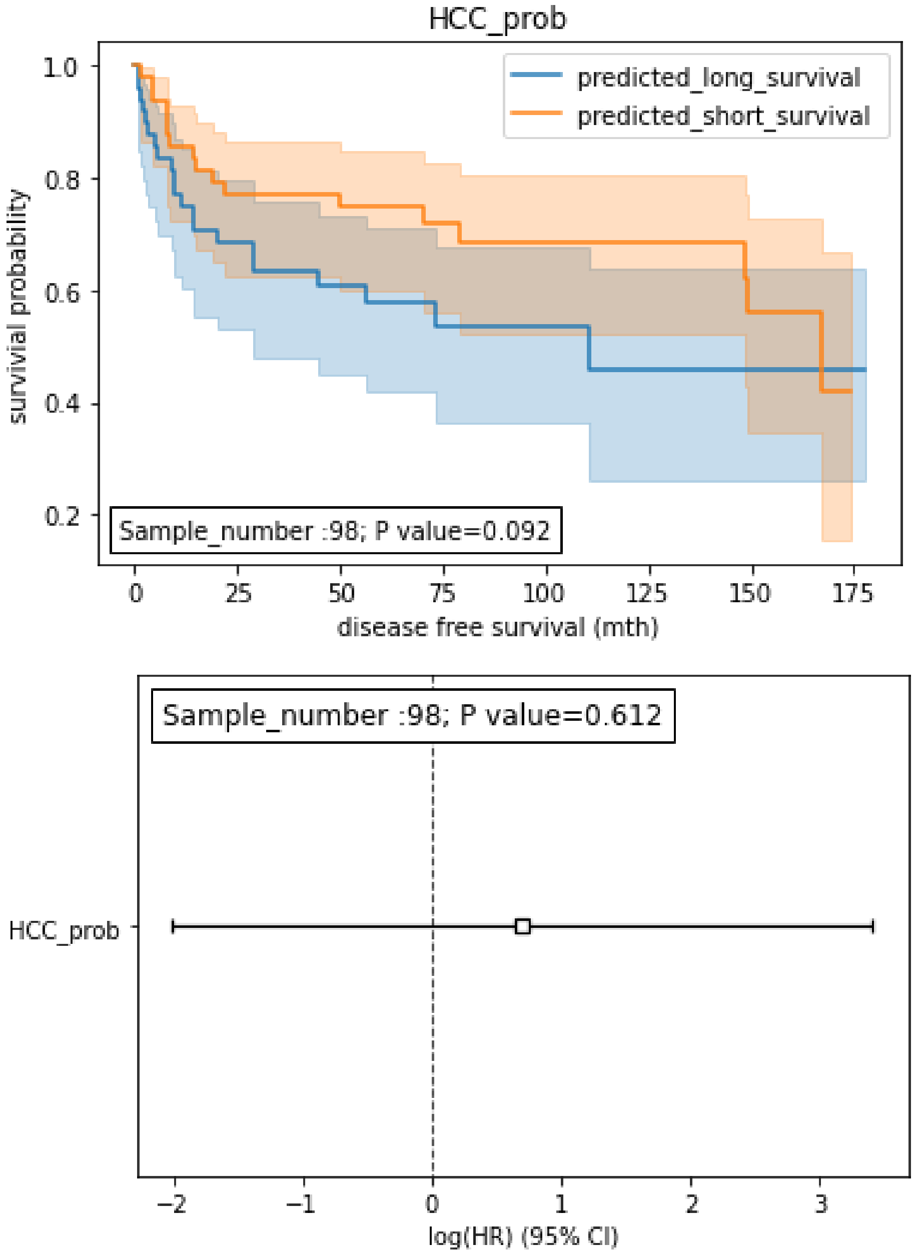

Supplementary Figure 3: Performance of HCCseek-23 panel for HCC disease-free survival (DFS) prognosis as analyzed by log-rank and univariate Cox hazards tests. Kaplan-Meier survival curves and Univariate Cox shows no significant differences between the predicted HCC probability (cut-off > 0.5) and DFS (log-rank p-value= 0.092, cox p-value – 0.612).

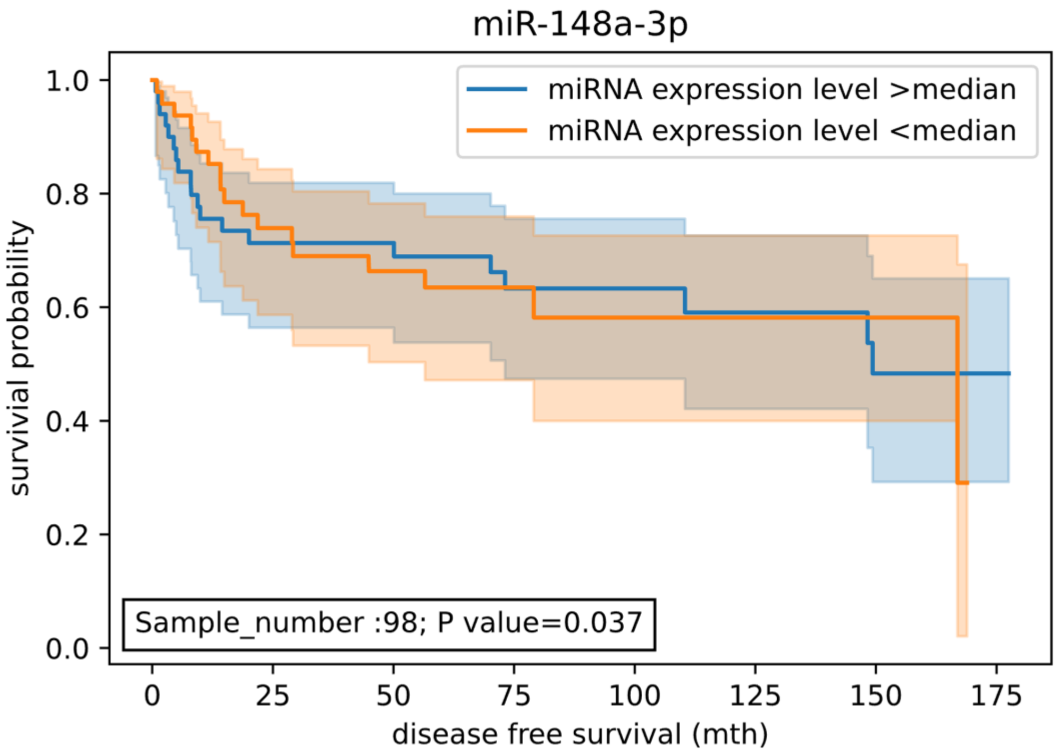

Supplementary Figure 4: KM analysis demonstrated a significant association between the miR-148a-3p expression before surgery and disease-free survival (p=0.037)

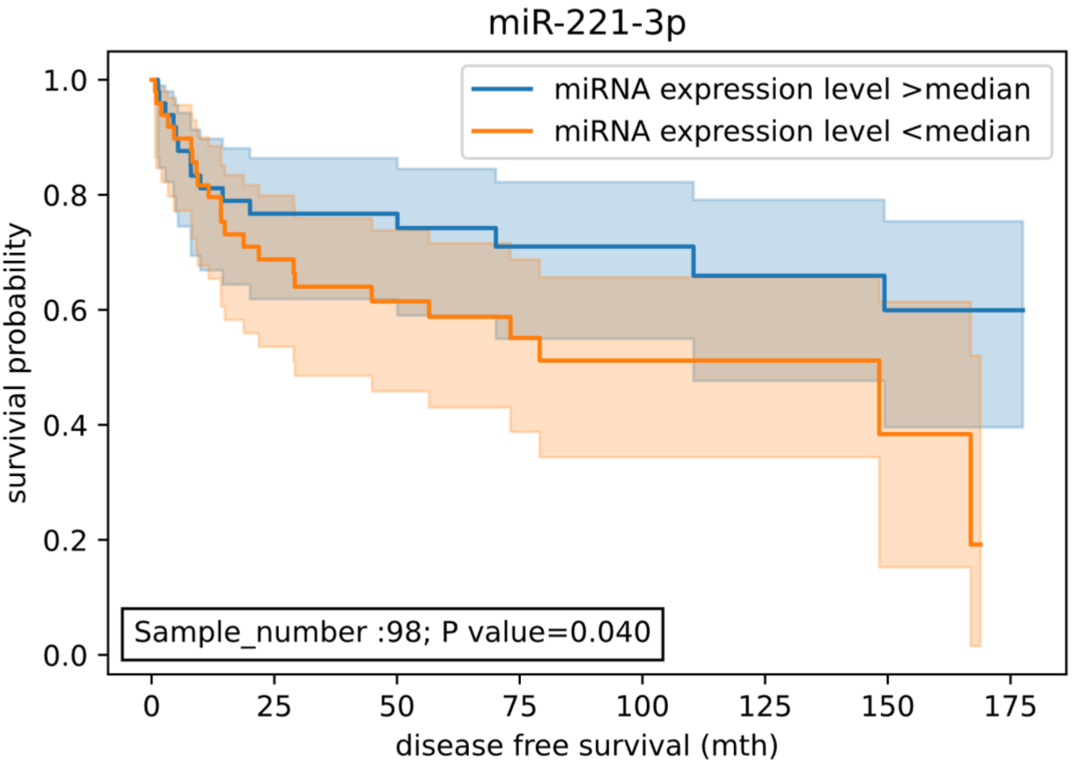

Supplementary Figure 5: KM analysis demonstrated a significant association between the miR-221-3p expression before surgery and disease-free survival (p=0.04)

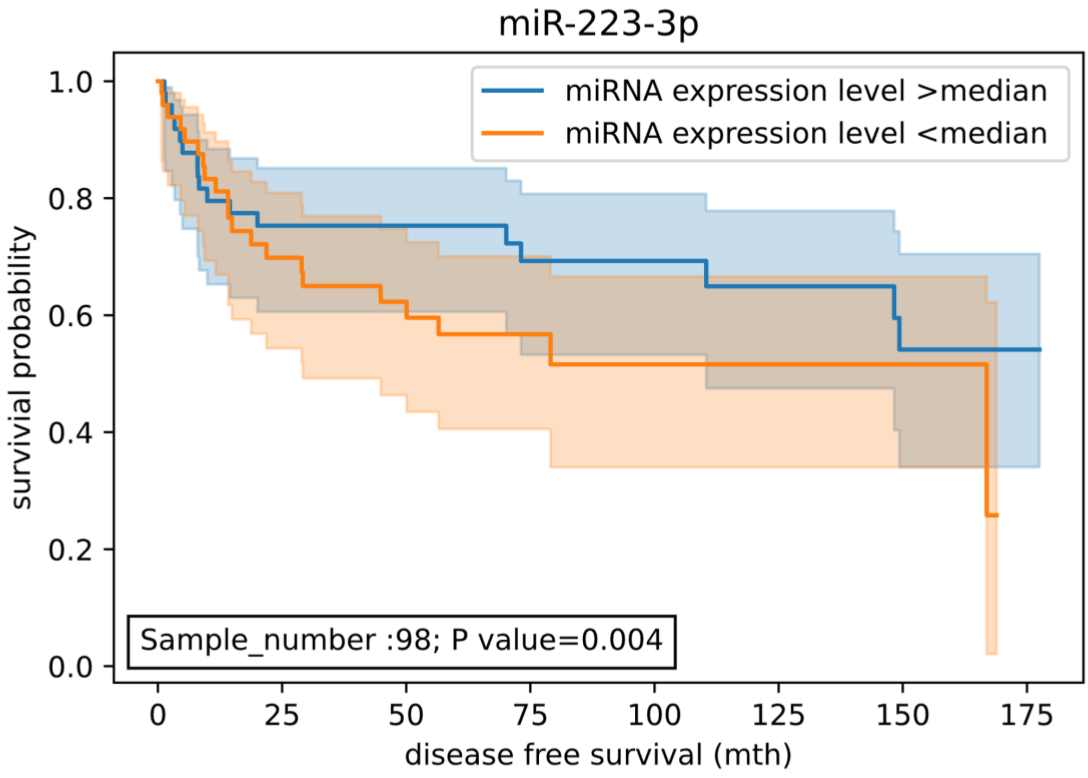

Supplementary Figure 6: KM analysis demonstrated a significant association between the miR-223-3p expression before surgery and disease-free survival (p=0.004)

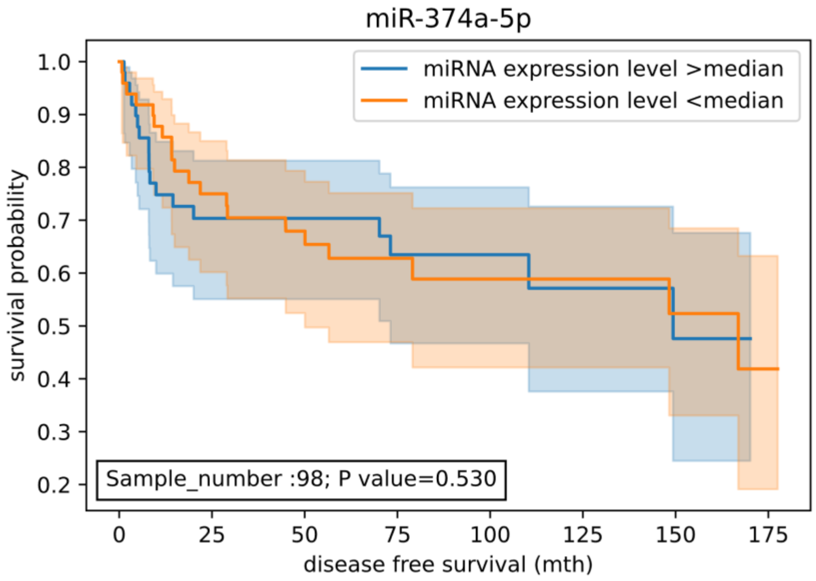

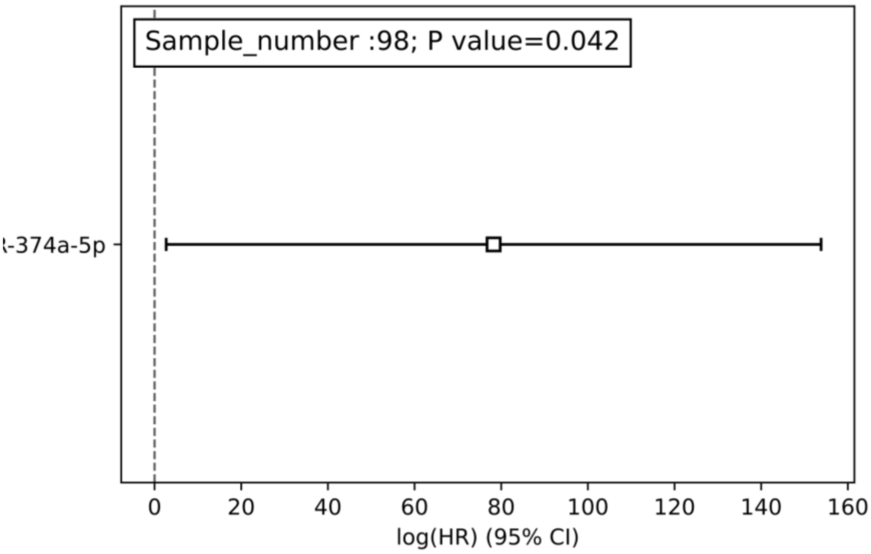

Supplementary Figure 7: KM and Cox proportional hazards analyses for miR-374a-5p. Cox proportional hazards analysis demonstrated a significant association between the miR-374a-5p expression before surgery and disease-free survival (Cox p-value =0.042)

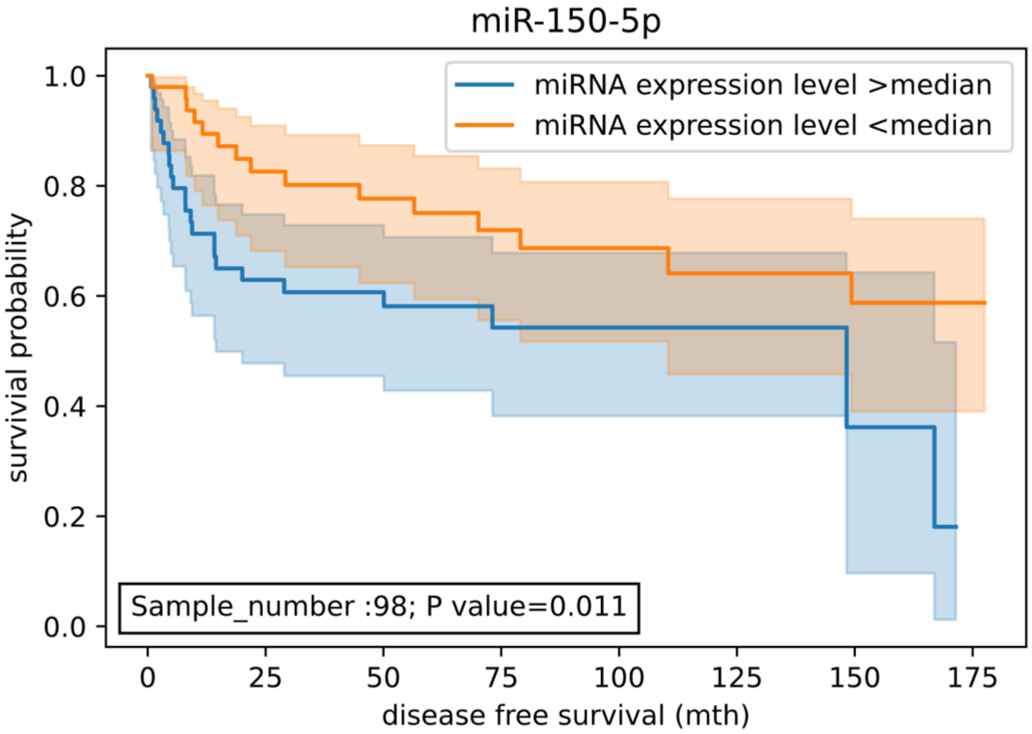

Supplementary Figure 8: KM analysis demonstrated a significant association between the miR-150-5p expression after surgery and disease-free survival (p=0.011)

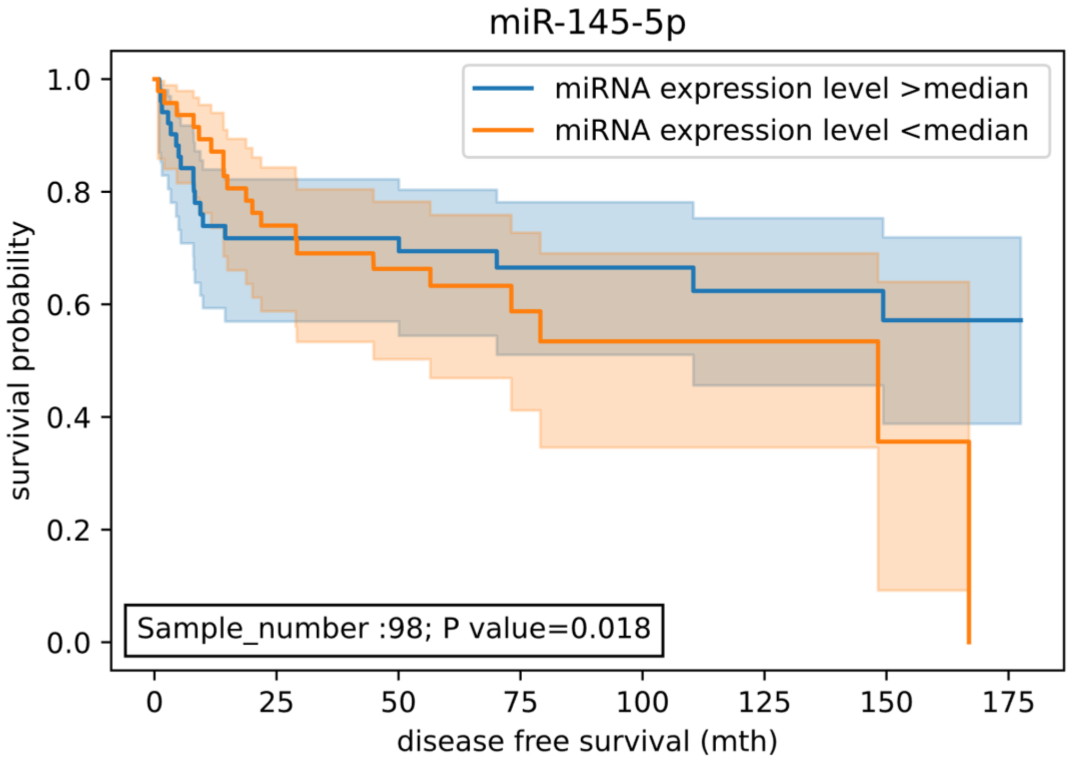

Supplementary Figure 9: KM analysis demonstrated a significant association between the differential expression level of miR-145-5p (B2-B1) and disease-free survival (p=0.018). B2 indicates miRNA expression level before surgery. B2 indicates miRNA expression level after surgery.

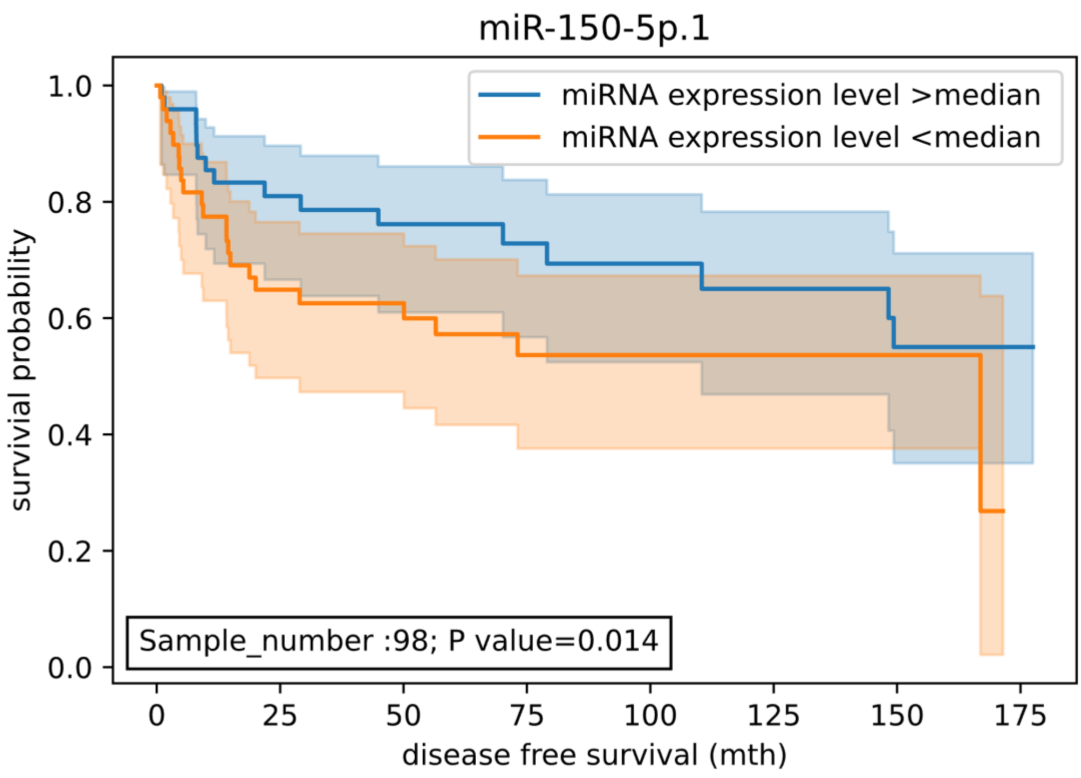

Supplementary Figure 10: KM analysis demonstrated a significant association between the differential expression level of miR-150-5p (B2-B1) and disease-free survival (p=0.014). B2 indicates miRNA expression level before surgery. B2 indicates miRNA expression level after surgery.

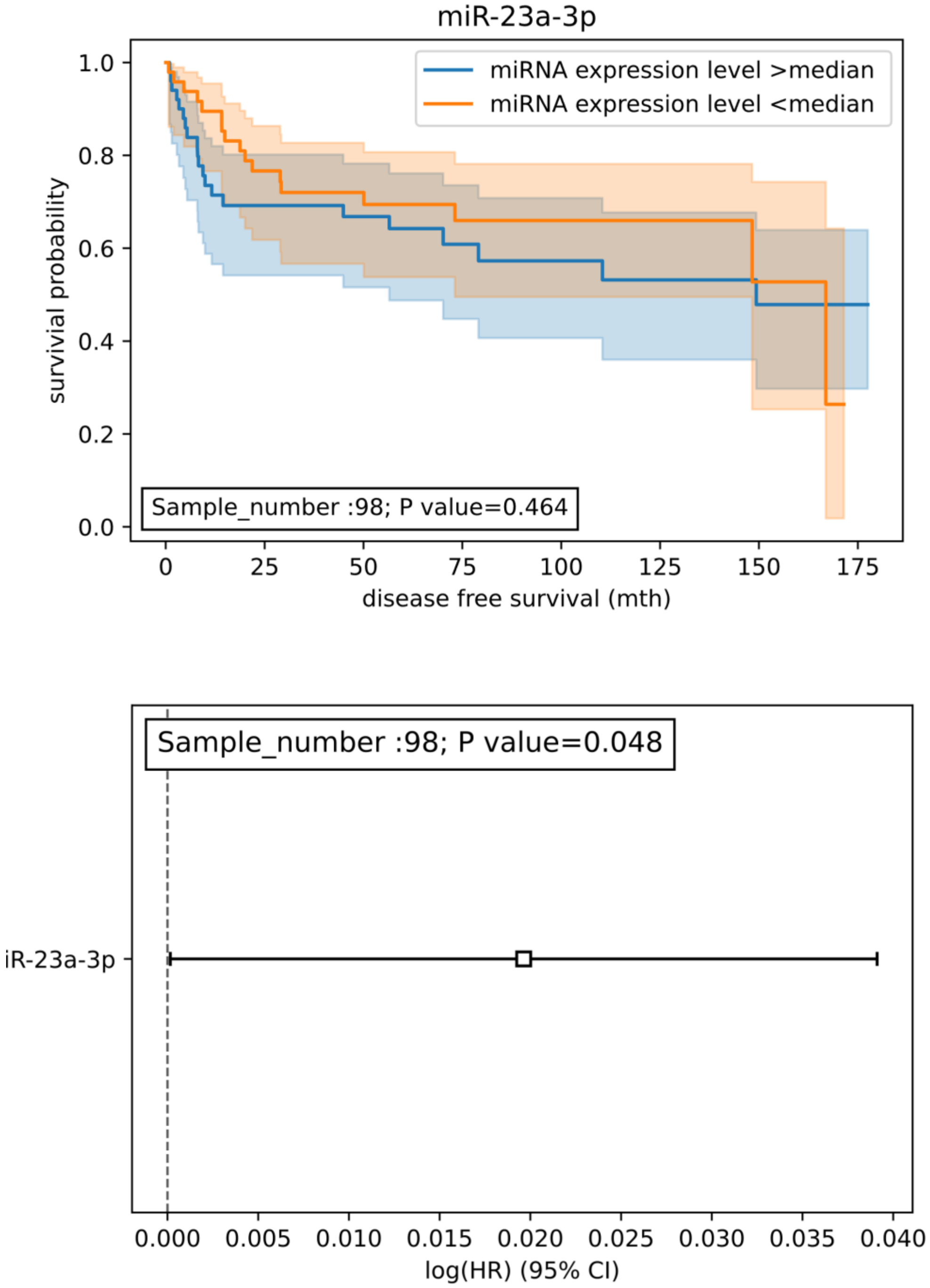

Supplementary Figure 11: KM and Cox proportional hazards analyses for of miR-23a-3p. Cox proportional hazards analysis demonstrated a significant association between the differential expression level of miR-23a-3p (B2-B1) and disease-free survival (p=0.048). B2 indicates miRNA expression level before surgery. B2 indicates miRNA expression level after surgery.

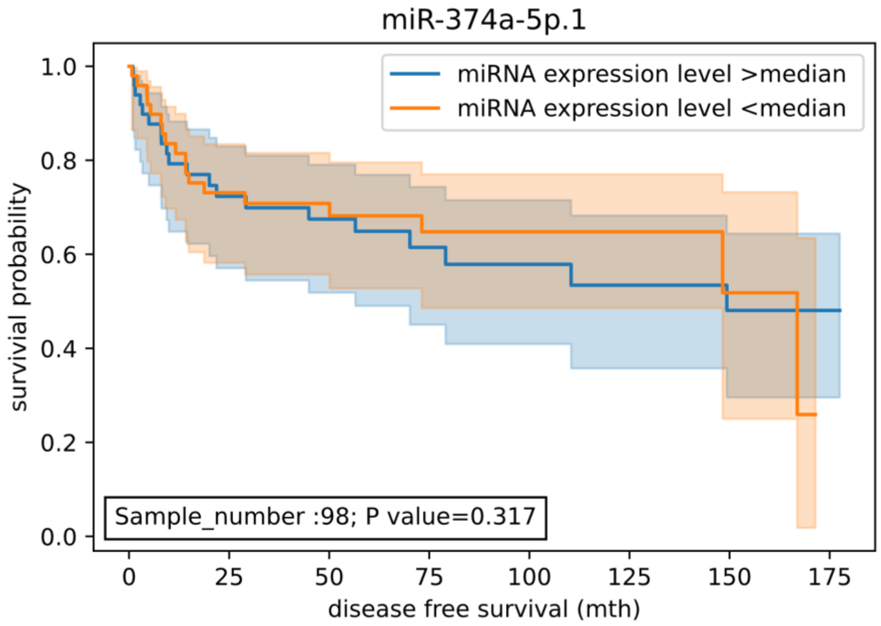

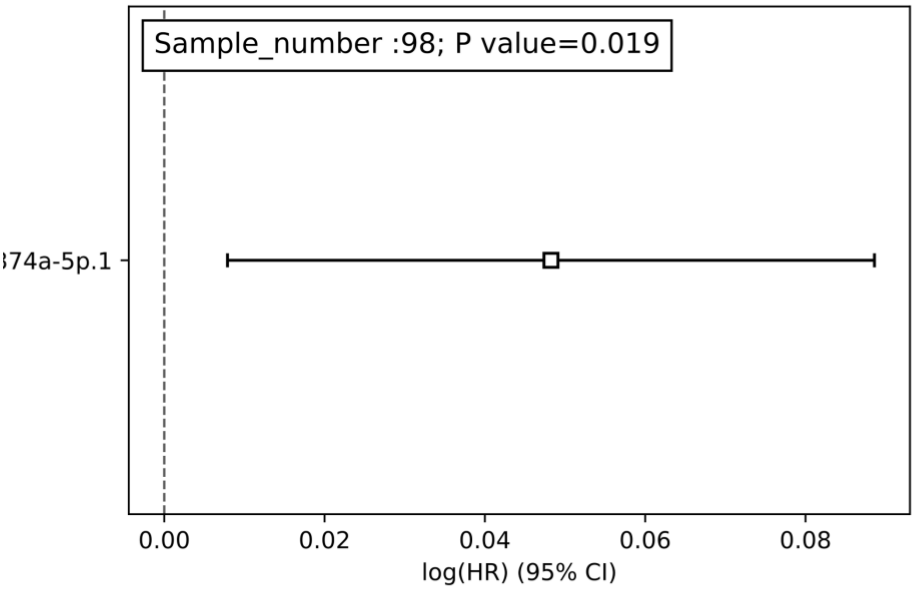

Supplementary Figure 12: KM and Cox proportional hazards analyses for of miR-374a-5p. Cox proportional hazards analysis demonstrated a significant association between the differential expression level of miR-374a-5p (B2-B1) and disease-free survival (p=0.019). B2 indicates miRNA expression level before surgery. B2 indicates miRNA expression level after surgery.

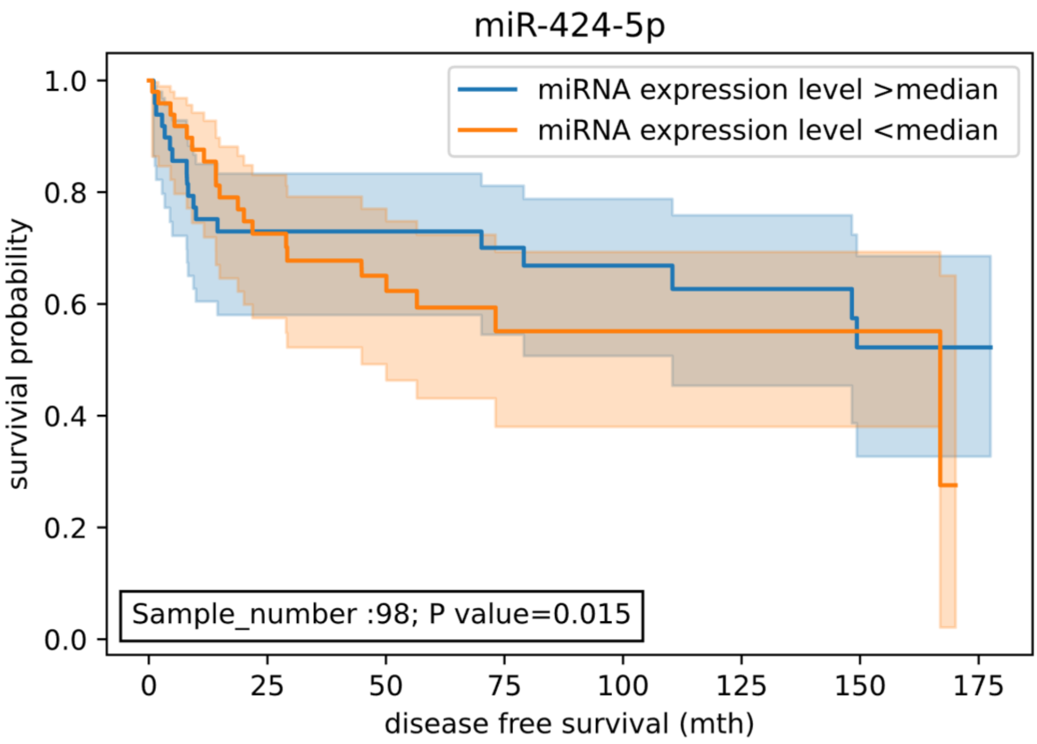

Supplementary Figure 13: KM analysis demonstrated a significant association between the differential expression level of miR-424-5p (B2-B1) and disease-free survival (p=0.015). B2 indicates miRNA expression level before surgery. B2 indicates miRNA expression level after surgery.

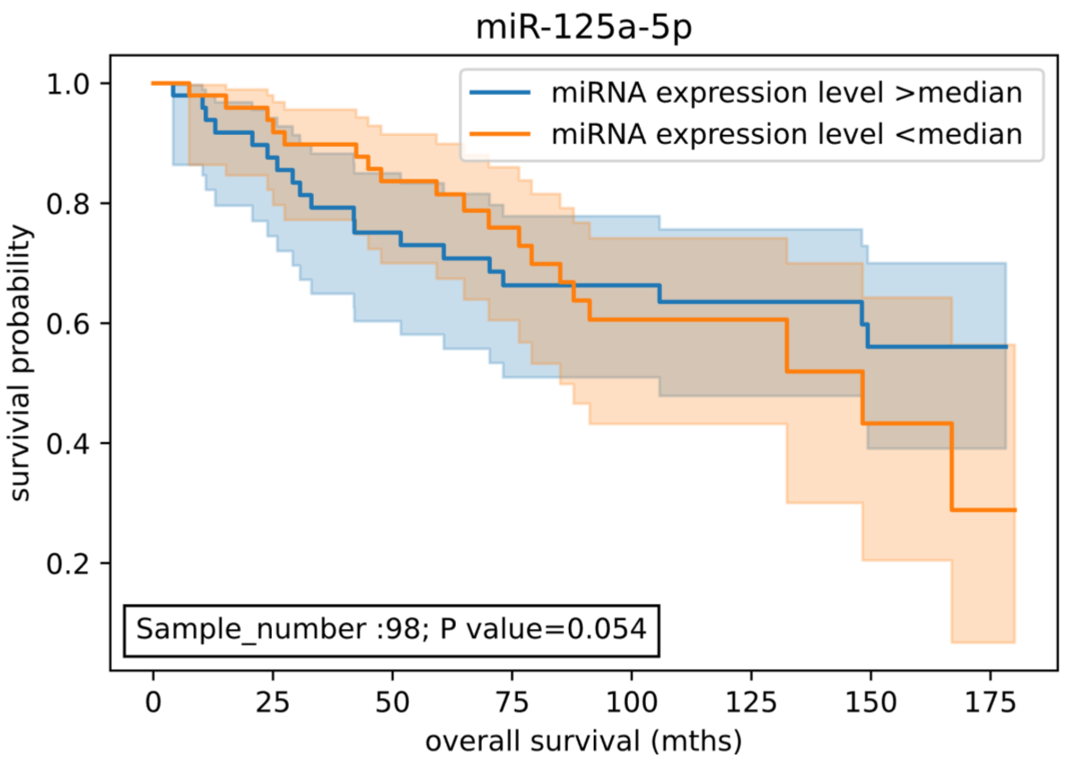

Supplementary Figure 14: KM analysis demonstrated a marginal association between the miR-125a-5p expression before surgery and overall survival (p=0.054)

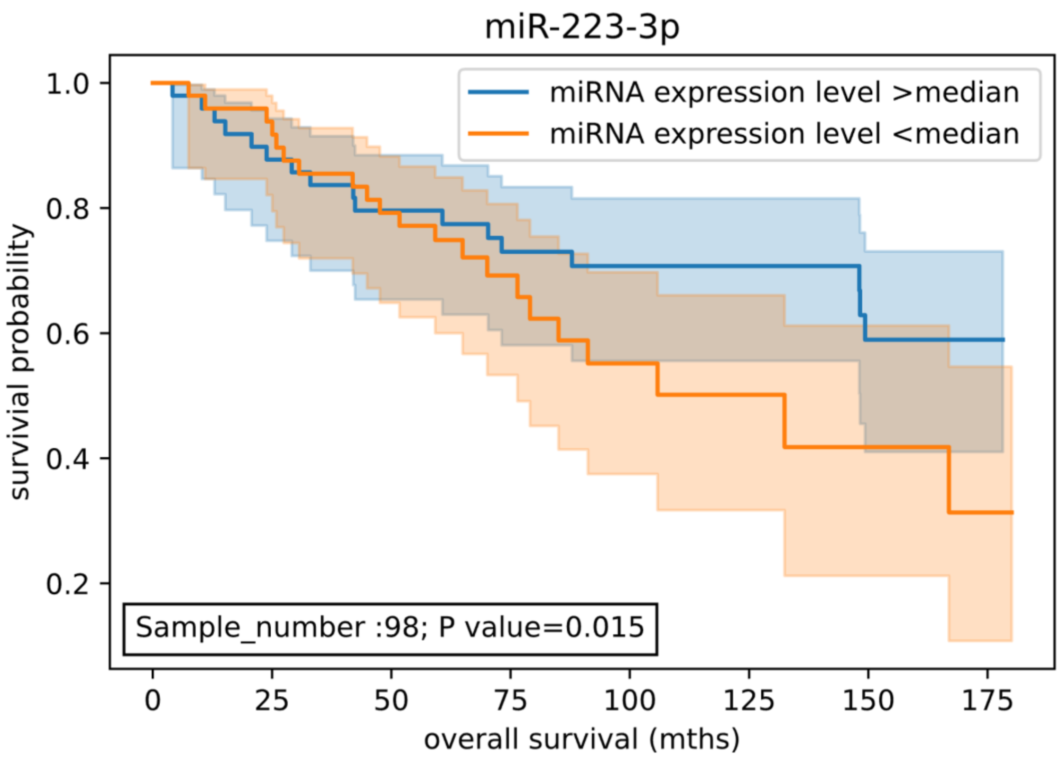

Supplementary Figure 15: KM analysis demonstrated a significant association between the miR-223-3p expression before surgery and overall survival (p=0.015)

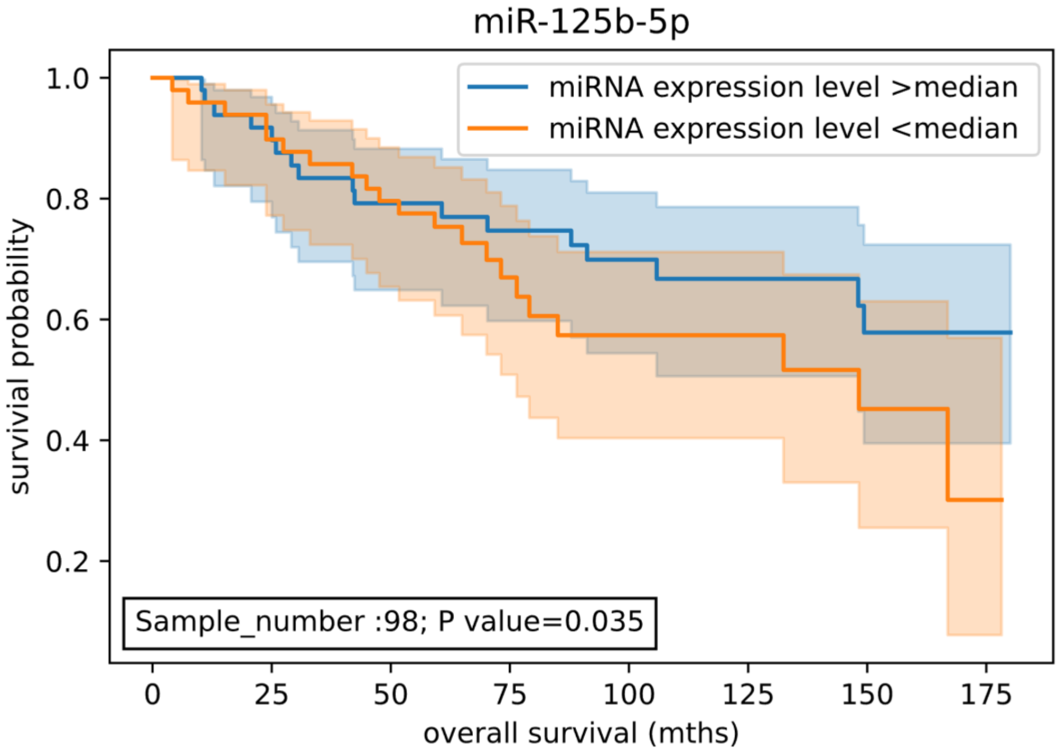

Supplementary Figure 16: KM analysis demonstrated a significant association between the differential expression level of miR-125b-5p (B2-B1) and overall survival (p=0.035). B2 indicates miRNA expression level before surgery. B2 indicates miRNA expression level after surgery.

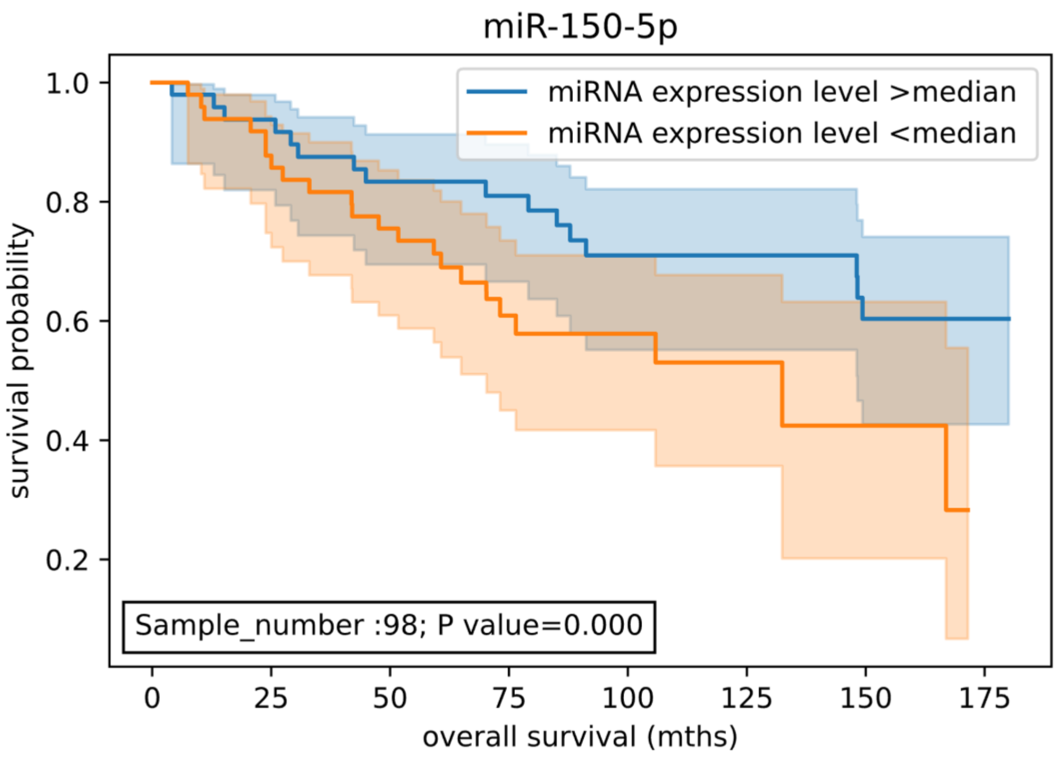

Supplementary Figure 17: KM analysis demonstrated a significant association between the differential expression level of miR-150-5p (B2-B1) and overall survival (p=0.000). B2 indicates miRNA expression level before surgery. B2 indicates miRNA expression level after surgery.

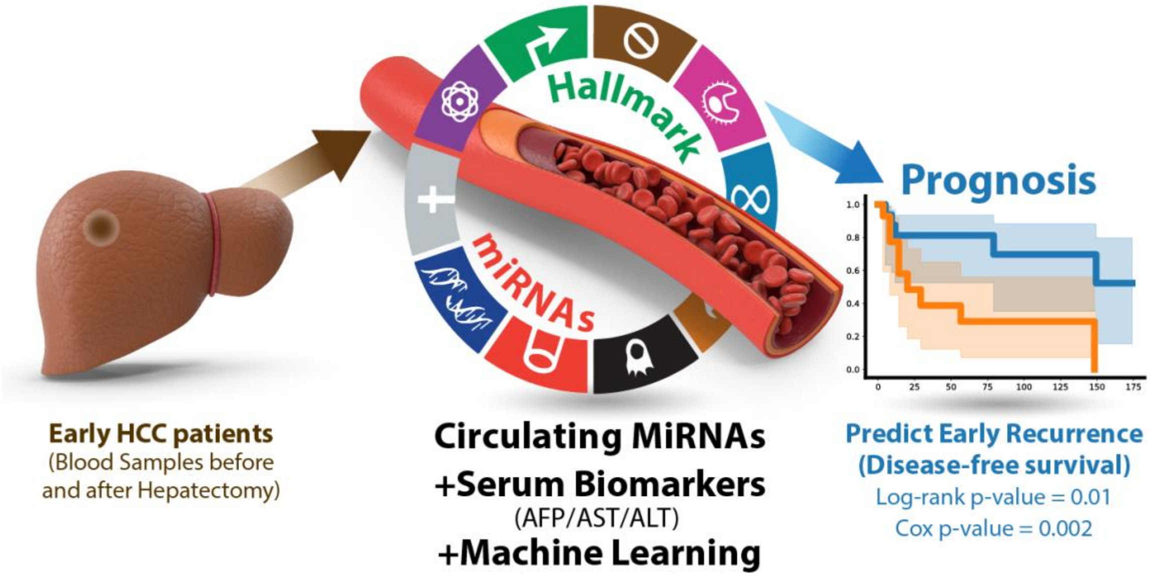

Supplementary Figure 18: Schematic diagram of this circulating microRNA study
